# Genome sequence of *Enterobacter vonholyi* H2G27 isolated from the gut of Bangladesh’s national fish, *Tenualosa ilisha*

**DOI:** 10.1128/mra.00318-26

**Published:** 2026-06-22

**Authors:** Fahim Al Galib, Abdullah Al Kafi, Soharth Hasnat, Tahsin Islam Sakif, M. Nazmul Hoque, Md Morshedur Rahman, Dipali Rani Gupta, Tofazzal Islam

**Affiliations:** 1 Institute of Biotechnology and Genetic Engineering (IBGE), Gazipur Agricultural University (GAU) 198780, Gazipur, Bangladesh; 2 West Virginia University 5631, Morgantown, West Virginia, USA; 3 Department of Gynecology, Obstetrics and Reproductive Health, GAU 198780, Gazipur, Bangladesh; 4 Department of Dairy and Poultry Science, Gazipur Agricultural University (GAU) 198780, Gazipur, Bangladesh; Loyola University Chicago, Chicago, Illinois, USA

**Keywords:** Oxford Nanopore sequencing, probiotic potential, aquaculture management, gut microbiome

## Abstract

The 4.7-Mbp complete genome of *Enterobacter vonholyi* strain H2G27, isolated from Bangladesh’s national fish, hilsa shad (*Tenualosa ilisha*), was assembled into two contigs. Analysis confirmed a nonpathogenic profile and identified an antimicrobial bottromycin biosynthetic gene cluster. These findings highlight the strain’s probiotic potential for sustainable aquaculture management.

## ANNOUNCEMENT

Members of the genus *Enterobacter* are core components of the fish gut microbiota. Microbiome studies of the hilsa shad, *Tenualosa ilisha*, from brackish and marine environments have shown this genus representing 15.81% and 5.74% of the microbial community, respectively (1, 2). Host associated isolates are considered promising probiotic candidates due to their capacity to tolerate gastrointestinal conditions, inhibit fish pathogens, and colonize the host gut (3, 4).

The isolate H2G27 was obtained from the gut of Bangladesh’s national fish, hilsa (*T. ilisha*), captured by local fishermen (gill net catch) from the Meghna River in Chandpur, Bangladesh (22.8333° N, 90.8333° E). Fishes were processed immediately after transport on ice. The gastrointestinal tract was aseptically (sterilization with 70% ethanol) removed and homogenized in sterile phosphate-buffered saline and plated (serially diluted) on nutrient agar (Oxoid, UK) at 37°C for 48 h (5). Distinct colonies were purified by streaking thrice (37°C for 24 h). A purified single colony was designated as strain H2G27 based on consistent colony morphology and a lack of hemolytic activity on a blood agar base (Oxoid). Gram staining and biochemical characterization identified the isolate as a Gram-negative, rod-shaped, catalase-positive, and oxidase-negative bacterium, consistent with the *Enterobacter* genus (6, 7).

Genomic DNA from an overnight (37°C) NA culture was extracted using the Tissue Genomic DNA Extraction HE Mini Kit (Favorgen, Taiwan) and quantified with a Qubit 4.0 fluorometer (Thermo Fisher Scientific). Libraries were prepared using the Oxford Nanopore Technologies (ONT) Ligation Sequencing Kit (SQK-NBD114.24) with the NEBNext Companion Module and Native Barcoding Kit 96 (v14) (no shearing and no size selection) and sequenced on an ONT MinION using an R10.4.1 flow cell (8). Basecalling with Dorado (v1.2.0) (SUP model) yielded 36,328 reads (N50, 15,202). The read quality was evaluated using NanoPlot (v1.46.1) (9), filtered (*Q* > 15, >1,000 bp length) using NanoFilt (v2.8.0) (9), *de novo* assembled using Flye (v2.9.5-b1801) (11), and polished with Racon (v1.5.0) (10) and Medaka (v2.0.1) (11). Publicly available genome annotation was performed using the NCBI PGAP (v6.10) (12). Assembly quality and completeness were assessed using QUAST (v5.2.0) (13) and BUSCO (v6.0.0) (*-l bacteria_odb10*, *-m genome*) (14). Additional functional features were predicted using PlasmidFinder (v2.0.1) (17) and the RAST (v2.0) server (15). Default parameters were used for all software unless otherwise specified.

GTDB-Tk (v2.6.1) (16) and NCBI ANI analyses (17) identified H2G27 as *Enterobacter vonholyi* with 98.07% ANI and 89.69% coverage against the type strain genome GCA_008364555.1. The gapless, unrotated assembly comprised one circular chromosome (4,564,296 bp) and one circular plasmid (134,708 bp) with 55.5% GC content, consistent with the Flye output, encoding 4,369 coding sequences and 116 RNA genes (Table 1). RAST analysis predicted 337 metabolic features, including subsystems for potassium-18, sulfur-24, iron acquisition-35, nitrogen-25, phosphorus-28, and stress response-91. Secondary metabolite analysis via antiSMASH (v8.0.4) (18) identified six biosynthetic gene clusters (BGCs), including terpene, NRP-enterobactin-like, RiPP, homoserine lactone, NI-siderophore, and arylpolyene clusters. PathogenFinder (v2.0) predicted a nonpathogenic profile (score: 0.4) (19), while BAGEL4 (20) identified an antimicrobial bottromycin BGC. These genomic features and its nonpathogenic profile highlight the probiotic potential of strain H2G27 for sustainable aquaculture, pending *in vivo* validation.

**TABLE 1:** Genomic features of *E. vonholyi* strain H2G27 isolated from the gut of hilsa

Genomic features	H2G27
GenBank accession no.	JBSVSI000000000
SRA accession no.	SRS28386804
Assembly accession no.	GCA_054403075.1
BioSample accession no.	SAMN53736890
Number of reads	36,328 (read N50, 15,202)
Assembled genome size (bp)	4,699,615 bp
GC content (%)	55.5
Average coverage (×)	40
Genome completeness (%)	99.2 (BUSCO)
Contamination (%)	0.2
N50 value (bp)	4,564,295
Number of contigs	2 (one chromosome and one plasmid)
Genes (total)	4,485
Coding sequences (CDSs)	4,369
Genes (coding)	4,315
CDSs (with protein)	4,315
Genes (RNA)	116
rRNAs	9, 8, 8 (5S, 16S, 23S)
Complete rRNAs	9, 8, 8 (5S, 16S, 23S)
tRNAs	83
ncRNAs	8
Pseudogenes	54
CDSs (without protein)	54
Pseudogenes (ambiguous residues)	0 of 54
Pseudogenes (frameshifted)	27 of 54
Pseudogenes (incomplete)	35 of 54
Pseudogenes (internal stop)	10 of 54
Pseudogenes (multiple problems)	16 of 54
Plasmid replicons	1
Number of subsystems (metabolic features)	337

## Data Availability

The whole-genome shotgun project of the *Enterobacter vonholyi* strain H2G27 has been deposited in GenBank under BioProject (Mining Bangladesh Biogold) accession number PRJNA1055760. The Sequence Read Archive accession number is SRS28386804; the BioSample accession number is SAMN53736890. The version described in this paper is version JBSVSI000000000. Additional data sets are available in Zenodo (https://doi.org/10.5281/zenodo.20261689).
